# A Comprehensive Review of Upper Gastrointestinal Symptom Management in Autoimmune Gastritis: Current Insights and Future Directions

**DOI:** 10.7759/cureus.43418

**Published:** 2023-08-13

**Authors:** Shubhangi Singh, Swarupa Chakole, Suyash Agrawal, Nidhi Shetty, Roshan Prasad, Tejaswee Lohakare, Mayur Wanjari, Seema Yelne

**Affiliations:** 1 Medicine, Jawaharlal Nehru Medical College, Datta Meghe Institute of Higher Education and Research, Wardha, IND; 2 Community Medicine, Jawaharlal Nehru Medical College, Datta Meghe Institute of Higher Education and Research, Wardha, IND; 3 Internal Medicine, Jawaharlal Nehru Medical College, Datta Meghe Institute of Higher Education and Research, Wardha, IND; 4 Child Health Nursing, Smt. Radhikabai Meghe Memorial College of Nursing, Datta Meghe Institute of Higher Education and Research, Wardha, IND; 5 Research and Development, Jawaharlal Nehru Medical College, Datta Meghe Institute of Higher Education and Research, Wardha, IND; 6 Nursing, Shalinitai Meghe College of Nursing, Datta Meghe Institute of Higher Education and Research, Wardha, IND

**Keywords:** precision medicine, dietary modifications, pharmacological interventions, diagnosis, symptom management, upper gastrointestinal symptoms, autoimmune gastritis

## Abstract

Autoimmune gastritis is characterized by inflammation of the gastric mucosa due to autoimmune dysregulation. Upper gastrointestinal symptoms associated with autoimmune gastritis can significantly impact an individual's quality of life and require effective management strategies. This review article provides a comprehensive overview of the current understanding of upper gastrointestinal symptom management in autoimmune gastritis, aiming to consolidate existing knowledge, identify gaps, and offer insights for future research and clinical practice. The review begins by discussing the background and significance of autoimmune gastritis, highlighting its prevalence and the impact of upper gastrointestinal symptoms on affected individuals. The pathophysiology and clinical presentation of autoimmune gastritis-related upper gastrointestinal symptoms are explored, emphasizing the need for accurate diagnosis and targeted management approaches. Diagnostic approaches, including diagnostic criteria, endoscopy, histology, and biomarkers, are critically examined, along with the challenges and limitations associated with diagnosing autoimmune gastritis. The review then delves into the pharmacological approaches for symptom relief, such as proton pump inhibitors (PPIs) and H2 receptor antagonists. It explores the role of dietary modifications and lifestyle changes in symptom control. The article further discusses recent advancements in pharmacological interventions, novel therapeutic approaches, and the potential benefits of complementary and alternative medicine in symptom management. The concept of patient-centered approaches and personalized management strategies is emphasized, highlighting the importance of considering individual patient characteristics, treatment goals, and preferences. Recommendations for future research and clinical management are provided, including exploring emerging therapeutic targets, precision medicine approaches, and collaboration among researchers, clinicians, and patient advocacy groups. The review concludes by emphasizing the significance of implementing the findings and recommendations in clinical practice to enhance patient care and improve the quality of life for individuals with autoimmune gastritis.

## Introduction and background

Autoimmune gastritis is a chronic inflammatory condition of the stomach characterized by the destruction of parietal cells and subsequent impairment in gastric acid secretion. This immune-mediated process is driven by the production of autoantibodies against the H+/K+-ATPase (proton pump) and intrinsic factor, leading to the malabsorption of vitamin B12 and causing pernicious anemia. Over time, the progressive loss of gastric acid-producing cells results in a hypo- or achlorhydric environment within the stomach [1-3].

Autoimmune gastritis represents a significant health concern, with an increasing prevalence worldwide. While the exact etiology remains unclear, genetic susceptibility and environmental factors are believed to play critical roles in its development. The disease often remains asymptomatic in its early stages, making diagnosis challenging until advanced stages manifest clinically [3,4]. Autoimmune gastritis has been associated with autoimmune disorders, such as type 1 diabetes, thyroid diseases, and celiac disease. This points toward its systemic nature and highlights the need for comprehensive management [5].

Upper gastrointestinal symptoms, including epigastric pain, heartburn, bloating, and early satiety, are among the primary complaints reported by patients with autoimmune gastritis. These symptoms significantly impact affected individuals' quality of life and daily functioning, necessitating timely and appropriate management [6]. Autoimmune gastritis is characterized by the presence of autoantibodies against the proton pump and intrinsic factor, leading to malabsorption of vitamin B-12 and pernicious anemia. However, it is crucial to recognize that the effects of this condition on the proton pump and reduced acid secretion may also result in the malabsorption of iron, causing iron-deficiency anemia [7]. These long-term complications can lead to severe health consequences if not promptly addressed.

This review article aims to provide a comprehensive overview of the management of upper gastrointestinal symptoms in patients with autoimmune gastritis. It aims to explore current insights into symptom relief strategies and their efficacy while discussing potential future directions for improving symptom management. This review will thoroughly examine the existing literature and focus on various pharmacological interventions, dietary modifications, and lifestyle changes utilized in the clinical setting. Furthermore, it will delve into recent advancements and emerging therapies that hold promise for improved symptom control.

## Review

Methodology

A comprehensive literature search was conducted using electronic databases, including PubMed, MEDLINE, and Google Scholar, to identify relevant studies for inclusion in this review article. The search strategy combined keywords and medical subject headings (MeSH terms) related to autoimmune gastritis, upper gastrointestinal symptoms, and symptom management. The search was limited to articles published in English and up to date. Reference lists of identified articles and relevant review papers were manually searched for additional studies. The inclusion criteria encompassed observational studies, clinical trials, systematic reviews, and meta-analyses focused on individuals diagnosed with autoimmune gastritis experiencing upper gastrointestinal symptoms. Studies reporting various management strategies were considered, including pharmacological interventions, dietary modifications, and lifestyle changes. Articles that did not meet the inclusion criteria or were duplicates, abstracts, letters, or conference proceedings were excluded. The selection process involved screening titles and abstracts, followed by a full-text assessment of potentially relevant studies. Any disagreements were resolved through consensus among the review authors. The methodology was designed to ensure the inclusion of high-quality studies that provided valuable insights into upper gastrointestinal symptom management in autoimmune gastritis.

Autoimmune gastritis: pathophysiology and clinical presentation

Overview of Autoimmune Gastritis and its Etiology

Autoimmune gastritis is a chronic autoimmune disorder characterized by the immune-mediated destruction of parietal cells in the gastric mucosa. The exact etiology of autoimmune gastritis remains complex and multifactorial, involving genetic and environmental factors [4].

Genetic predisposition plays a significant role in the development of autoimmune gastritis. Certain human leukocyte antigen (HLA) alleles, particularly HLA-DR and HLA-DQ, have been associated with an increased risk of developing the condition. Genetic factors related to immune dysregulation and autoimmunity, such as specific gene polymorphisms, may also contribute to disease susceptibility [8].

Environmental factors, including infections (e.g., Helicobacter pylori) and exposure to certain dietary components, may trigger the autoimmune response in genetically susceptible individuals. In particular, molecular mimicry, where microbial or dietary antigens resemble self-antigens, may activate autoreactive immune cells targeting the gastric mucosa [9,10].

Pathophysiological Mechanisms Underlying Upper Gastrointestinal Symptoms

The pathophysiological mechanisms underlying upper gastrointestinal symptoms in autoimmune gastritis involve the loss of parietal cells and subsequent alterations in gastric acid secretion. Parietal cells are responsible for producing and secreting gastric acid, which aids in digestion and acts as a protective barrier against ingested pathogens [11].

As autoimmune gastritis progresses, the destruction of parietal cells leads to decreased or absent gastric acid secretion, resulting in hypochlorhydria or achlorhydria. This alteration in gastric acid levels disrupts the normal digestive processes and can contribute to upper gastrointestinal symptoms such as epigastric pain, heartburn, and bloating [12]. Furthermore, the destruction of parietal cells and the subsequent decrease in intrinsic factor production can impair the absorption of vitamin B12. Vitamin B12 deficiency, known as pernicious anemia, can cause additional symptoms such as fatigue, weakness, and neurological manifestations.

Clinical Presentation of Autoimmune Gastritis-Related Upper Gastrointestinal Symptoms

The clinical presentation of autoimmune gastritis-related upper gastrointestinal symptoms can vary among individuals. Some patients may remain asymptomatic or have nonspecific symptoms, especially in the early stages of the disease. However, as the condition progresses, the following symptoms may manifest.

Epigastric pain: Epigastric pain refers to a specific type of pain experienced in the upper abdomen, between the ribcage and the navel. In autoimmune gastritis, patients often report a dull, gnawing, or burning pain in this region. The pain can vary in intensity and may be described as discomfort or heaviness. It can occur intermittently or persistently and may be triggered or worsened by certain foods or when the stomach is empty [13].

Heartburn: Autoimmune gastritis can contribute to the development of gastroesophageal reflux disease (GERD), characterized by the backward flow of stomach acid into the esophagus. This reflux can cause a burning sensation in the chest and throat, commonly known as heartburn. Patients with autoimmune gastritis may experience heartburn, particularly after meals or when lying down, as these positions can exacerbate the reflux [14].

Bloating and abdominal distension: Impaired digestion and motility in autoimmune gastritis can lead to symptoms of bloating and abdominal distension. Patients may feel a sense of fullness and increased gassiness, causing their abdomen to appear and feel distended. These symptoms can be distressing and impact daily activities [15].

Early satiety refers to feeling full quickly after consuming only small amounts of food. This symptom is commonly reported in autoimmune gastritis and can significantly affect a person's eating habits and nutritional status. The feeling of fullness occurs prematurely during meals, leading to reduced food intake. Consequently, unintended weight loss may occur due to inadequate calorie consumption [16].

Other symptoms: In addition to the upper gastrointestinal symptoms mentioned above, autoimmune gastritis can be associated with symptoms related to vitamin B12 deficiency. The autoimmune attack on the gastric parietal cells can impair the production of intrinsic factors, a protein necessary to absorb vitamin B12. As a result, individuals may experience symptoms such as fatigue, weakness, neurological abnormalities (e.g., numbness, tingling, balance problems), and signs of anemia (e.g., pale skin, shortness of breath, rapid heartbeat). These symptoms often reflect the systemic effects of vitamin B12 deficiency and can significantly impact a person's overall well-being [17].

Understanding the clinical presentation of autoimmune gastritis-related upper gastrointestinal symptoms is crucial for accurately diagnosing and appropriately managing affected individuals.

Diagnostic approaches in autoimmune gastritis

Diagnostic Criteria for Autoimmune Gastritis

Diagnosing autoimmune gastritis involves a combination of clinical evaluation, endoscopic findings, histological examination, and specific laboratory tests. Various diagnostic criteria have been proposed, including the Sydney classification, the updated Operative Link for Gastritis Assessment (OLGA) staging system, and the recent recommendations by the World Health Organization (WHO) [18].

The diagnostic criteria typically encompass the presence of chronic gastritis with specific histopathological features, including lymphoplasmacytic infiltrates, glandular atrophy, and the absence of H. pylori infection. Additionally, the detection of autoantibodies, such as anti-parietal cell antibodies (APCA) and anti-intrinsic factor antibodies (AIFA), further supports the diagnosis of autoimmune gastritis [19].

Role of Endoscopy, Histology, and Biomarkers in Diagnosis

Endoscopy: Upper gastrointestinal endoscopy allows direct visualization of the gastric mucosa and can aid in assessing the severity and extent of gastric inflammation and atrophy. Endoscopic findings suggest autoimmune gastritis includes mucosal erythema, nodularity, and atrophic changes. However, it is important to note that endoscopic findings alone are insufficient for a definitive diagnosis and should be combined with histological evaluation [20].

Histology: Histological examination of gastric biopsy samples remains the gold standard for diagnosing autoimmune gastritis. The characteristic features include chronic lymphoplasmacytic infiltrates, glandular atrophy, and loss of parietal cells. The presence of lymphoid follicles and the evaluation of the OLGA staging system can provide additional information on disease progression and the risk of developing gastric neoplasms [21].

Biomarkers: Several laboratory biomarkers can aid in the diagnosis of autoimmune gastritis. Autoantibodies, including APCA and AIFA, are commonly detected in affected individuals. Serum gastrin levels are also elevated due to the loss of negative feedback on gastrin release in the setting of reduced gastric acid secretion. Additionally, measure serum vitamin B12 levels to assess the deficiency and identify associated pernicious anemia [22].

Challenges and Limitations in Diagnosing Autoimmune Gastritis

Subclinical and early-stage disease: Autoimmune gastritis can present with subclinical or nonspecific symptoms in its early stages, making it challenging to diagnose. Asymptomatic individuals or those with mild, non-specific symptoms may not seek medical attention, resulting in delayed diagnosis and treatment initiation. Additionally, the histological features of autoimmune gastritis can overlap with other forms of chronic gastritis, such as Helicobacter pylori-associated gastritis, further complicating the diagnostic process [23].

Sampling errors: The patchy distribution of autoimmune gastritis within the stomach poses a challenge in obtaining accurate biopsy samples during endoscopy. The autoimmune inflammation may be localized to specific areas, and sampling errors can occur if biopsies are taken from unaffected regions. Multiple biopsies from different gastric regions are often necessary to improve diagnostic accuracy and increase the likelihood of detecting characteristic histological features [24].

Interpretation of histological findings: Histological examination of gastric biopsies plays a crucial role in diagnosing autoimmune gastritis. However, interpreting the histological findings requires expertise, and there can be interobserver variability. Pathologists with experience in recognizing the specific histological features of autoimmune gastritis, such as lymphocytic infiltrates, glandular atrophy, and metaplasia, are essential for accurate diagnosis [25].

False-negative serological tests: Serological tests for autoantibodies, such as APCA and AIFA, are valuable diagnostic markers for autoimmune gastritis. However, false-negative results can occur, particularly in the early stages of the disease or in patients with atypical serological profiles. Relying solely on serological tests may lead to missed diagnoses, highlighting the importance of a comprehensive diagnostic approach that includes clinical evaluation, endoscopy, histology, and consideration of other relevant factors [26].

Overlapping comorbidities: Autoimmune gastritis frequently coexists with other autoimmune disorders, such as celiac disease and type 1 diabetes. The presence of multiple autoimmune conditions can complicate the diagnostic process as symptoms may be attributed to one specific condition, masking the underlying autoimmune gastritis. Clinicians must be aware of these overlapping comorbidities and perform a thorough evaluation to ensure an accurate diagnosis and appropriate management [27]. Despite these challenges, clinical evaluation, endoscopic assessment, histological examination, and serological testing remain crucial in diagnosing autoimmune gastritis and differentiating it from other forms of chronic gastritis.

Upper gastrointestinal symptom management in autoimmune gastritis

Pharmacological Approaches for Symptom Relief

Proton pump inhibitors (PPIs) and their efficacy: PPIs are commonly prescribed medications for managing upper gastrointestinal symptoms in autoimmune gastritis. They reduce gastric acid secretion by inhibiting the H+/K+ ATPase pump and alleviate symptoms such as heartburn and epigastric pain. The progressive loss of gastric acid-producing cells in autoimmune gastritis can result in a hypo- or achlorhydric environment within the stomach, leading to potential malabsorption issues. For instance, reduced acid secretion can significantly impact the absorption of certain nutrients, such as iron, due to impaired conversion of dietary iron to its absorbable form. Consequently, patients with autoimmune gastritis may experience iron malabsorption due to the effects of this condition on the proton pump and reduced acid secretion. While PPIs have demonstrated efficacy in improving symptoms and promoting the healing of gastric mucosal lesions, it's important to note that they may not fully address all symptoms, especially those related to functional dyspepsia and bloating [28,29]. Thus, a comprehensive management approach that considers the potential malabsorption issues and addresses other symptoms may be necessary to treat autoimmune gastritis [28,29].

H2 receptor antagonists and their role: H2 receptor antagonists, such as ranitidine and famotidine, can also manage upper gastrointestinal symptoms. They work by reducing gastric acid production, providing symptomatic relief for conditions like heartburn and acid reflux. While they are generally less potent than PPIs, they can be considered alternative options for symptom control in patients with autoimmune gastritis [30].

Other medications for symptom management: Additional medications may be prescribed depending on the specific symptoms and individual patient needs. For example, prokinetic agents like metoclopramide can enhance gastric emptying and reduce symptoms of bloating and early satiety. Antacids and alginate-based preparations may temporarily relieve symptoms by neutralizing gastric acid or forming a protective barrier in the stomach [31].

Dietary Modifications and Their Impact on Symptom Control

Avoidance of trigger foods: Certain foods can exacerbate symptoms in individuals with autoimmune gastritis. Common triggers include spicy and fatty foods, citrus fruits, caffeine, alcohol, and carbonated beverages. Patients are advised to identify and avoid specific trigger foods to minimize symptom flare-ups. This involves careful observation and keeping a food diary to track symptom triggers. By eliminating or reducing the consumption of these trigger foods, individuals can experience relief from symptoms such as abdominal pain, bloating, and indigestion [32].

Small, frequent meals: Consuming smaller, more frequent meals rather than large, heavy meals can help alleviate symptoms such as bloating and early satiety. This approach allows for better digestion and reduces the strain on the stomach. By spacing out meals throughout the day, individuals can prevent overloading the stomach, improving digestion and reducing symptoms. Focusing on nutrient-dense, easily digestible foods during these smaller meals is important for adequate nourishment [33].

Low-fat and low-acid diet: A low-fat diet can help reduce the workload on the digestive system, while a low-acid diet can minimize acid reflux symptoms. This particularly benefits individuals with autoimmune gastritis who may experience increased stomach acid production. By reducing the consumption of high-fat foods, such as fried and greasy foods, individuals can avoid triggering symptoms such as acid reflux, heartburn, and nausea. Similarly, limiting the intake of acidic foods, such as tomatoes, citrus fruits, and vinegar, can help reduce stomach acid production and alleviate associated symptoms. Emphasizing whole grains, lean proteins, fruits, and vegetables can provide a balanced and nourishing diet while minimizing symptom exacerbation [34].

Lifestyle Changes and Their Role in Symptom Management

Weight management: Maintaining a healthy weight is crucial for managing upper gastrointestinal symptoms in conditions like autoimmune gastritis. Excess weight can pressure the stomach, leading to heartburn and acid reflux symptoms. By adopting healthy eating habits, engaging in regular physical activity, and achieving a healthy body weight, individuals can alleviate the burden on their stomachs and experience relief from these symptoms [35].

Stress reduction: Stress and anxiety have been known to exacerbate gastrointestinal symptoms, including those associated with autoimmune gastritis. Implementing stress reduction techniques can significantly improve symptom control. Practices such as mindfulness meditation, deep breathing exercises, and regular physical activity can help individuals manage stress levels, promote relaxation, and positively impact their gastrointestinal health. By incorporating these techniques into their daily routine, individuals may experience a reduction in the frequency and severity of upper gastrointestinal symptoms [36].

Smoking cessation: Smoking has been linked to an array of adverse effects on gastrointestinal health, including increased acid production and impaired healing of the gastric mucosa. Individuals with autoimmune gastritis should consider quitting smoking to improve symptom management and overall health. Smoking cessation reduces the risk of complications associated with autoimmune gastritis and helps alleviate symptoms such as heartburn, acid reflux, and gastritis flare-ups. By quitting smoking, individuals can promote the healing of the gastric mucosa and enhance the effectiveness of other management strategies [37].

Healthcare providers must work closely with patients to develop an individualized approach to symptom management, considering the specific symptom profile, underlying disease severity, and the patient's preferences and response to various treatment modalities. Regular follow-up and adjustments to the treatment plan may be necessary to achieve optimal symptom control and improve the quality of life for individuals with autoimmune gastritis.

Current insights in upper gastrointestinal symptom management

Recent Advancements in Pharmacological Interventions

Mucosal protective agents: Sucralfate and other mucosal protective agents have emerged as potential therapeutic options for managing upper gastrointestinal symptoms in autoimmune gastritis. These agents work by forming a protective barrier on the gastric mucosa, reducing inflammation, and promoting healing. Their ability to adhere to the ulcerated mucosa provides a physical barrier against gastric acid and other irritants, relieving symptoms and enhancing mucosal recovery [38].

Ghrelin receptor agonists: Ghrelin, a hormone primarily produced in the stomach, regulates appetite and gastric motility. Ghrelin receptor agonists, such as tesamorelin, are being investigated as potential therapies for improving upper gastrointestinal symptoms in autoimmune gastritis. By stimulating ghrelin receptors, these agonists aim to enhance gastric emptying, alleviate symptoms of early satiety and postprandial fullness, and potentially improve overall gastrointestinal function [39].

Immunomodulatory therapies: Given the immune dysregulation observed in autoimmune gastritis, immunomodulatory therapies are being explored as potential treatment options. These therapies target specific immune pathways involved in the pathogenesis of autoimmune gastritis. T-cell modulators, such as immunosuppressive agents or immune checkpoint inhibitors, aim to regulate abnormal T-cell responses. At the same time, B-cell depletion therapies specifically target B cells involved in the autoimmune response. Other biologics targeting specific inflammatory mediators, such as cytokines or chemokines, are being investigated for their potential to modulate the immune response and reduce inflammation in autoimmune gastritis [40].

Novel Therapeutic Approaches and Their Potential Benefits

Gut microbiota modulation: Emerging evidence suggests that alterations in the gut microbiota may play a role in autoimmune gastritis. The imbalance of microbial communities in the gut can contribute to immune dysregulation and inflammation in the gastric mucosa. Strategies that modulate the gut microbiota, such as probiotics, prebiotics, and fecal microbiota transplantation, are being explored as potential therapeutic interventions. By restoring a healthy balance of gut bacteria, these approaches promise to improve symptoms and restore gut homeostasis in autoimmune gastritis [41].

Neuro-immune interactions: The bidirectional communication between the gut and the central nervous system, known as the gut-brain axis, plays a crucial role in gastrointestinal function and symptom perception. In autoimmune gastritis, aberrant neuro-immune interactions may contribute to the development and persistence of upper gastrointestinal symptoms. Therapies targeting neuro-immune interactions are being investigated as potential approaches for symptom relief. These may include neuromodulators, which modulate neural signaling in the gut, and neurokinin receptor antagonists, which block the activity of certain neurotransmitters involved in symptom generation. By targeting the intricate interplay between the nervous and immune systems, these therapies can potentially improve upper gastrointestinal symptoms in autoimmune gastritis [42].

Targeted therapies: Advances in understanding the molecular mechanisms underlying autoimmune gastritis have opened up opportunities for targeted therapies. Researchers have identified specific immune targets or pathways involved in the autoimmune response, presenting potential targets for therapeutic intervention. Targeted therapies selectively modulate these specific immune components to suppress the autoimmune response and alleviate symptoms. Examples of targeted therapies being explored in autoimmune gastritis include immune checkpoint inhibitors, monoclonal antibodies against specific cytokines or immune cells, and small molecules that modulate key immune signaling pathways. By specifically addressing the underlying immunological abnormalities in autoimmune gastritis, targeted therapies offer the potential for more precise and effective symptom management [43].

Role of Complementary and Alternative Medicine in Symptom Relief

Complementary and alternative medicine (CAM) encompasses diverse therapeutic approaches often used alongside conventional medical treatments. In autoimmune gastritis, CAM approaches have gained attention for their potential role in managing upper gastrointestinal symptoms. While the evidence supporting their use in autoimmune gastritis is limited, studies conducted in related gastrointestinal conditions suggest potential benefits in symptom relief and improving overall well-being [44].

One commonly explored CAM approach is acupuncture, which involves the insertion of thin needles at specific points in the body to stimulate healing and balance energy flow. While research on acupuncture in autoimmune gastritis is limited, studies in other gastrointestinal disorders, such as functional dyspepsia and irritable bowel syndrome, have shown promising results in reducing symptoms such as abdominal pain, bloating, and nausea [45].

Herbal medicine, another CAM component, involves using plant-based preparations and extracts to manage symptoms and promote health. While specific herbal remedies for autoimmune gastritis may not have been extensively studied, certain herbs and botanicals have been traditionally used for gastrointestinal conditions. For example, peppermint oil has been found to alleviate symptoms of dyspepsia and facilitate gastric emptying, potentially offering relief for individuals with autoimmune gastritis experiencing similar symptoms [46].

Mind-body techniques, including relaxation exercises, meditation, and yoga, focus on connecting the mind and body to promote overall well-being. While the evidence directly linking these practices to autoimmune gastritis is limited, research in other chronic conditions, such as inflammatory bowel disease, suggests that mind-body techniques can reduce stress, improve quality of life, and potentially alleviate gastrointestinal symptoms [47].

It is important to note that while CAM approaches may offer potential benefits, their use should be cautiously discussed with healthcare providers. Integrating CAM therapies with conventional treatments is essential to ensure safety, prevent potential interactions or adverse effects, and optimize overall treatment outcomes. Healthcare providers can provide guidance and help individuals make informed decisions regarding using CAM as adjunctive therapy for symptom relief in autoimmune gastritis [48].

Patient-Centered Approaches and Personalized Management Strategies

Patient-centered approaches and personalized management strategies in autoimmune gastritis acknowledge the heterogeneity of symptoms and treatment responses among affected individuals. It recognizes that each patient is unique and requires an individualized approach to their care [49].

In a patient-centered approach, healthcare professionals consider various factors when developing a management plan. These factors include the specific symptom profile experienced by the patient, their treatment goals, preferences, and any potential comorbidities or coexisting conditions they may have. By considering these individual factors, healthcare providers can tailor the management strategies to address each patient's specific needs and circumstances [50].

Shared decision-making is an integral part of patient-centered care. It involves active participation and open communication between the healthcare provider and the patient, allowing the patient to be involved in the decision-making process regarding their treatment options. This collaborative approach ensures that the patient's preferences and values are considered and respected, leading to a more personalized and patient-centric management plan [51].

Regular communication between the healthcare provider and the patient is crucial in patient-centered care. This allows for ongoing assessment of treatment response, monitoring of symptoms, and adjustment of the management plan as needed. By maintaining a continuous dialogue with the patient, healthcare providers can evaluate the effectiveness of the chosen interventions and make necessary modifications to optimize symptom control and improve the patient's overall quality of life [52].

Overall, patient-centered approaches and personalized management strategies in autoimmune gastritis prioritize each patient's individual needs and preferences. By taking into account the unique characteristics and circumstances of the patient, healthcare providers can develop tailored treatment plans that address the individual's specific symptomatology and treatment goals, leading to improved outcomes and enhanced patient satisfaction.

Future directions and emerging therapies

Promising Areas of Research in Autoimmune Gastritis Symptom Management

Microbiota-targeted therapies: The gut microbiota plays a crucial role in autoimmune gastritis, and further research is needed to understand its specific contribution. Microbiota-targeted therapies aim to restore a healthy microbial balance in the gut and alleviate symptoms. Personalized probiotic formulations, tailored to an individual's specific microbiota composition, may be developed to promote beneficial bacterial strains and enhance gut homeostasis. Another approach involves microbial metabolite supplementation, which provides specific metabolites produced by the gut microbiota to promote a favorable environment and modulate the immune response in autoimmune gastritis [53].

Immunomodulation strategies: The immune system is intricately involved in the pathogenesis of autoimmune gastritis. Immunomodulatory therapies have shown promise in managing upper gastrointestinal symptoms by modulating the immune response and reducing inflammation. Immune checkpoint inhibitors, which target specific immune regulatory molecules, cytokine modulators that regulate the production and activity of pro-inflammatory cytokines, and targeted immunotherapies that selectively modulate immune cell populations, are potential avenues for symptom management in autoimmune gastritis. These approaches aim to restore immune balance, reduce autoimmune-mediated damage, and alleviate symptoms [54].

Neuro-immune interactions: The gut-brain axis and neuro-immune interactions are crucial in gastrointestinal function and symptom perception. Continued research into this complex interplay may unveil novel therapeutic targets for symptom management in autoimmune gastritis. Understanding how neural and immune signaling pathways intersect can provide insights into symptom generation mechanisms. Targeting specific neurotransmitter receptors or neural pathways involved in gastrointestinal function may lead to the development of more targeted and effective therapies, improving symptom control and overall patient outcomes. By addressing the intricate connections between the nervous and immune systems, innovative therapeutic approaches may be developed to alleviate upper gastrointestinal symptoms in autoimmune gastritis [55].

Potential Targets for Novel Therapies

Cytokines and inflammatory mediators: Targeting specific cytokines, such as tumor necrosis factor-alpha (TNF-α), interleukin-17 (IL-17), or other key inflammatory mediators, holds promise as a potential therapeutic approach in autoimmune gastritis. These cytokines play a crucial role in the inflammatory response within the gastric mucosa. By selectively blocking or modulating their activity, it may be possible to alleviate symptoms and modify the course of the disease. Further research is needed to investigate the effectiveness and safety of targeting specific cytokines in autoimmune gastritis [56].

Autoantibodies and B-cell pathways: Autoimmune gastritis is characterized by autoantibodies targeting specific components of the gastric mucosa. Investigating the role of autoantibodies and B-cell pathways in autoimmune gastritis can provide insights into the underlying mechanisms driving the disease. Targeting these autoantibodies or modulating B-cell activity may offer novel therapeutic approaches to address the immune response in autoimmune gastritis specifically. Developing therapies that specifically target these components of the immune response holds the potential to improve symptom control and potentially slow down disease progression [57].

Neurotransmitter receptors and neural pathways: The gastrointestinal tract is regulated by a complex network of neurotransmitter receptors and neural pathways that influence gut motility, sensation, and symptom perception. Identifying specific neurotransmitter receptors and modulating neural pathways involved in gastrointestinal function can open new avenues for managing upper gastrointestinal symptoms in autoimmune gastritis. Targeting these receptors or pathways may be possible to modulate gut motility, reduce visceral hypersensitivity, and alleviate symptoms. However, further research is needed to understand better the intricate interplay between the immune and nervous systems in autoimmune gastritis and identify the most effective targets for therapeutic intervention [58].

Precision Medicine and Tailored Treatments for Symptom Control

The concept of precision medicine, which involves tailoring treatments to individual patients based on their unique characteristics, is gaining traction in autoimmune gastritis symptom management. Some potential approaches in this direction include:

Biomarker-based treatment strategies: Biomarkers are measurable indicators that can provide valuable information about a patient's health status or response to treatment. Identifying specific biomarkers associated with symptom severity or treatment response in autoimmune gastritis can have significant clinical implications. Clinicians can personalize treatment decisions by analyzing biomarkers, such as specific proteins or genetic markers. Biomarker-based treatment strategies can help predict individual treatment outcomes, guide the selection of appropriate interventions, and optimize symptom management in autoimmune gastritis [59].

Genomic and genetic factors: Understanding genomic and genetic factors underlying autoimmune gastritis is an active research area. Gene mutations or single nucleotide polymorphisms (SNPs) may play a critical role in the development and progression of autoimmune gastritis and individual responses to treatment. By identifying specific genetic variants associated with symptom profiles or treatment responses, clinicians can gain valuable insights into the underlying mechanisms of the disease and develop targeted therapies or personalized treatment strategies. Notably, one area of interest is exploring the impact of these genetic variations on the proton pump and reduced acid secretion, which may lead to malabsorption of iron as a consequence. Exploring the interplay of genomic and genetic factors in relation to iron absorption could potentially shed light on novel approaches for managing autoimmune gastritis and optimizing treatment outcomes [60]. Combining genomic and genetic factors opens up promising avenues for precision medicine in managing autoimmune gastritis [60].

Multidimensional symptom assessment: Traditional approaches to symptom assessment often focus on a single symptom or its intensity. However, autoimmune gastritis and its associated upper gastrointestinal symptoms are complex and multifaceted. To effectively manage symptoms, it is crucial to consider their frequency, intensity, impact on daily life, and associated psychological factors. A comprehensive evaluation of these multidimensional aspects of symptoms enables healthcare professionals to tailor treatment plans to address each patient's specific needs. By adopting a multidimensional symptom assessment approach, clinicians can provide more holistic and patient-centered care for individuals with autoimmune gastritis [6].

Future perspectives and challenges in the field

Long-term monitoring: Long-term monitoring refers to conducting studies or observational research over an extended period to evaluate emerging therapies' long-term effects and safety in autoimmune gastritis. These studies aim to assess the durability of treatment response, potential side effects, and impact on disease progression. By monitoring patients over an extended period of time, researchers and clinicians can gain valuable insights into novel therapies' effectiveness and safety profile, enabling evidence-based decision-making for long-term management [61].

Individual variability: Autoimmune gastritis exhibits significant variability among individuals regarding symptom presentation and treatment response. Understanding the factors contributing to this variability is crucial for developing personalized treatment approaches. Genetic variations, immune system profiles, gut microbiota composition, and environmental influences may play a role in determining an individual's response to therapies. By identifying these factors, healthcare professionals can tailor treatment plans to suit each patient's specific needs, optimizing symptom control and improving treatment outcomes [62].

Collaboration and data sharing: Collaboration among researchers, clinicians, and patient advocacy groups is essential for advancing the field of autoimmune gastritis symptom management. Collaborative efforts facilitate the exchange of knowledge, resources, and expertise, leading to accelerated progress in understanding the disease and developing effective treatment strategies. Additionally, data sharing enables the pooling of information from multiple sources, enhancing the statistical power and generalizability of research findings. Standardized outcome measures and multicenter studies help generate robust evidence, establishing best practices for managing autoimmune gastritis symptoms [63].

Access to novel therapies: Ensuring access to emerging therapies for all individuals with autoimmune gastritis is a critical challenge. While novel therapies hold promise in improving symptom control and patient outcomes, barriers such as affordability, availability, and equitable distribution can hinder widespread implementation. It is crucial to address these barriers and ensure that individuals with autoimmune gastritis have equal opportunities to benefit from the latest advancements in treatment. Efforts to improve affordability, increase the availability of therapies, and promote equitable distribution can help overcome access-related challenges and improve the overall management of autoimmune gastritis symptoms [64]. By addressing these challenges and embracing emerging research opportunities, autoimmune gastritis symptom management can continue to advance, leading to more effective and personalized treatment options for individuals with this condition.

## Conclusions

Autoimmune gastritis is a complex condition characterized by inflammation of the gastric mucosa due to autoimmune dysregulation. Upper gastrointestinal symptoms associated with autoimmune gastritis can significantly impact an individual's quality of life and require effective management strategies. This comprehensive review has provided valuable insights into the current understanding of upper gastrointestinal symptom management in autoimmune gastritis, highlighting key findings and implications for clinical practice. The review emphasized the importance of accurate diagnosis, considering the diagnostic criteria for autoimmune gastritis and utilizing various diagnostic approaches, including endoscopy, histology, and biomarkers. Challenges and limitations in diagnosing autoimmune gastritis were acknowledged, underscoring the need for a multidimensional diagnostic approach. Pharmacological approaches, such as proton pump inhibitors (PPIs) and H2 receptor antagonists, were discussed as mainstays of symptom relief. Additionally, dietary modifications and lifestyle changes were explored as adjunctive strategies for symptom control. The review also highlighted recent advancements in pharmacological interventions, novel therapeutic approaches, and the role of complementary and alternative medicine in symptom management. The implications for clinical practice are significant. Healthcare professionals should be aware of the impact of upper gastrointestinal symptoms in autoimmune gastritis and consider a comprehensive approach to diagnosis and management. Individualized treatment plans, tailored to the patient's symptom profile, treatment goals, and preferences, are crucial. Shared decision-making and ongoing assessment of treatment response should guide clinical management.
